# Co-incidence of Human Papillomaviruses and Epstein–Barr Virus Is Associated With High to Intermediate Tumor Grade in Human Head and Neck Cancer in Syria

**DOI:** 10.3389/fonc.2020.01016

**Published:** 2020-08-20

**Authors:** Ishita Gupta, Lina Ghabreau, Hamda Al-Thawadi, Amber Yasmeen, Semir Vranic, Ala-Eddin Al Moustafa, Mohammed I. Malki

**Affiliations:** ^1^College of Medicine, QU Health, Qatar University, Doha, Qatar; ^2^Biomedical Research Centre, Qatar University, Doha, Qatar; ^3^Pathology Department, Faculty of Medicine, University of Aleppo, Aleppo, Syria; ^4^Syrian Research Cancer Centre of the Syrian Society Against Cancer, Aleppo, Syria; ^5^Segal Cancer Centre, Lady Davis Institute for Medical Research of the Sir Mortimer B. Davis-Jewish General Hospital, Montreal, QC, Canada

**Keywords:** head and neck cancers, human papillomaviruses, Epstein–Barr virus, tumor grade, Syrian population

## Abstract

High-risk human papillomaviruses (high-risk HPVs) have been recently reported to be co-present with Epstein–Barr virus (EBV) in different types of human cancers including head and neck (HN), where they can cooperate in the initiation and/or progression of this cancer. Accordingly, we herein explored the prevalence of high-risk HPVs and EBV in 80 HN cancer tissues from the Syrian population using polymerase chain reaction, immunohistochemistry, and tissue microarray methodologies. We report that high-risk HPVs and EBV are present in 35/80 (43.7%) and 41/80 (51.2%) of our samples, respectively, and the most frequent HPV types are 33, 16, 18, 45, 52, 58, 35, 51, and 31, in this order. More significantly, our data reveal that 25/80 (31.2%) of cancer cases are positive for high-risk HPVs as well as EBV, and their co-presence is associated with high/intermediate-grade squamous cell carcinomas. These data confirm the co-presence of high-risk HPVs and EBV in HN cancers in the Syrian population of the Middle East and demonstrate that their co-incidence is linked to a more aggressive cancer phenotype. Thus, future studies are required to confirm these data and elucidate the exact role of high-risk and EBV cooperation in human HN carcinogenesis.

## Introduction

Head and neck (HN) cancer is a broad term that incorporates epithelial malignancies located in the paranasal sinuses, oral cavity, nasal cavity, pharynx, and larynx (1). HN cancer is one of the most common among both male and female worldwide, with around 650,000 new cases and 330,000 deaths each year assessed by the World Health Organization (2); notably, most of these deaths occur in developing countries (3). When it comes to cancer-related mortality, it is generally either directly attributed to metastasis, as in tumor involvement of critical organs, or caused indirectly due to therapeutic resistance and the adverse effect of treatment on human organs (4, 5).

Today, it is well-known that more than 20% of human cancers are estimated to be linked with microorganism infections including oncoviruses infection especially high-risk human papillomaviruses (high-risk HPV) and Epstein–Barr virus (EBV) (6–8). More specifically, it has been well-established that high-risk HPV infections are critical etiological factors in the development of human HN cancers, especially oral, as ~40% of oral cancer cases are positive for high-risk HPVs, particularly types 16, 18, 31, 33, 35, 45, 52, and 58 worldwide including the Middle East (ME) region (7, 9). Additionally, it was pointed out that their presence is linked with vascular invasion and lymph node metastases in different types of human carcinomas including cervical and HN (10–12).

Likewise, EBV is a human gamma herpesvirus that commonly infects more than 90% of the adult population (13). Persistent infection with EBV can cause infectious mononucleosis, and its latent state can lead to several types of human B-cell lymphomas and certain solid cancers, especially nasopharyngeal (14–17); additionally, EBV has been shown to be strongly associated with undifferentiated nasopharyngeal carcinomas (NPCs). Several studies have detected the presence of EBV in HN squamous cell tumors implying its possible role in the development of malignancies throughout the upper aerodigestive tract (7, 18, 19). Moreover, it has been recently revealed that EBNA1 and LMP1 of EBV oncoproteins can enhance invasion of human cancer cells via the induction of epithelial-to-mesenchymal transition (EMT) (20, 21).

On the other hand, several recent studies revealed that high-risk HPVs and EBV are co-present in human HN cancers especially oral (22–24). Moreover, it has been reported that the co-occurrence of high-risk HPVs and EBV in oral cancer is associated with a significant increase in the invasiveness ability of cancer cells (25). We recently demonstrated that the co-presence of high-risk HPVs and EBV is linked to high/intermediate grade in different types of human carcinomas including HN (5, 26, 27). Thus, it is evident that the co-presence of high-risk HPVs and EBV in high-grade human carcinomas could suggest a possible cooperation between their oncoproteins; however, there are only few studies regarding the co-presence of high-risk HPVs and EBV in the ME region focusing only on NPCs.

Therefore, in this investigation, we assessed the presence of high-risk HPVs and EBV and their association with tumor phenotype in human HN cancer samples from Syria. Our study pointed out that high-risk HPVs and EBV are present in 43.7 and 51.2% of our samples, respectively, while co-incidence of these oncoviruses is 32.2%. More significantly, we noted that the co-incidence of these oncoviruses is associated with high/intermediate-grade squamous cell carcinomas in the majority of positive cases.

## Materials and Methods

### High-Risk HPV and EBV Detection

Eighty formalin-fixed paraffin-embedded blocks of HN cancer (57 larynx, 19 lower lip, 3 upper lip, and 1 nasopharynx) from Syrian patients, 73 males and 7 females, with an average age of 54.51 years were used. The samples were obtained from the Pathology Department, Faculty of Medicine of Aleppo University, Syria. Tissue blocks and data used in this report were approved, in March 22, 2009, by the Ethics Committee of the Faculty of Medicine of Aleppo University, # 2009-007, Aleppo, Syria. One hundred nanograms of DNA was extracted from each sample using Qiagen GmbH kit (Hilden, Germany). These samples were analyzed for high-risk HPVs and EBV by PCR using primers for E6/E7 genes of high-risk HPV types (16, 18, 31, 33, 35, 45, 51, 52, and 58) in addition to primers for LMP1 and EBNA1 genes of EBV; meanwhile, primers for the GAPDH gene were utilized as an internal control (26, 28). This analysis was achieved as illustrated earlier by our group (5, 26, 28).

### Tissue Microarray

Tissue microarray (TMA) building was realized as elucidated previously by our group (28, 29). Briefly, HN cancer samples were inserted into a virgin paraffin TMA block using a non-automated tissue arrayer (Beecher Instruments, Silver Spring, MD) irrespective of pathological staging information. Three TMA cores of 1.0 mm in diameter were sampled from a cohort of 80 block tissue samples of Syrian HN cancer patients. Afterwards, to verify the histological diagnosis, 4-μm sections were cut and stained with hematoxylin and eosin (H&E). Then, slides of the completed blocks were used for immunohistochemistry assay.

### Immunohistochemistry

Immunohistochemical (IHC) procedures examining the expression patterns of E6 and LMP1, of HPV and EBV, were done using standard practices. Briefly, slides were deparaffinized in graded alcohol, rehydrated, and boiled in 10 mM citrate buffer (pH 6.0) for antigen retrieval. Then, TMA slides were incubated for 35 min at 37°C with primary monoclonal and polyclonal antibodies for E6 of HPV and LMP1 of EBV (clones 1–4 and clone C1P5 from Dako and Calbiochem, Canada, respectively) using an automated immunostainer (Ventana Medical System, Tuscon, AZ). Afterwards, staining procedures were achieved according to the manufacturer's recommendations as slides were counterstained with hematoxylin prior to mounting. Negative controls were achieved by omitting primary antibody for E6 and LMP1. Following immunohistochemistry, two independent observers examined all TMA slides. The tumors were considered positive for E6 and LMP1 oncoproteins if cancer cells exhibited positivity ≥1% at any intensity (≥1+, scale 0–3+).

All TMAs also contained various cores representing positive and negative controls (e.g., cervical carcinoma and lymphatic tissues served as positive controls for HPV and EBV stains, respectively; normal HN tissues and epithelium were used as negative controls).

### Statistical Analysis

Statistical assessments were achieved using IBM SPSS Statistics (version 22; SPSS Inc., Chicago, IL, USA) and R. Data were analyzed as non-parametric files. We used χ^2^-test with Yates correction to explore the significance of the association between tumor grade and the co-incidence of high-risk HPVs and EBV.

## Results

In order to classify the presence of high-risk HPVs and EBV in human HN cancer tissues in the ME region, we explored the incidence of high-risk HPV types 16, 18, 31, 33, 35, 45, 51, 52, and 58 in a cohort of 80 HN cancer specimens from the Syrian population by PCR analysis, using specific primers for E6/E7 and LMP1 as well as EBNA1 genes of HPVs and EBV, respectively (5, 26, 28). Our data revealed that 35 (43.7%) and 41 (51.2%) of the 80 cancer samples are positive for high-risk HPVs and EBV, respectively (Table 1), and all of HPVs-positive specimens are co-infected with more than one HPV type. Regarding high-risk HPVs genotyping in these samples, our results pointed out that the most prevalent high-risk HPVs among the positive samples are types 33 (34/80), 16 (31/80), 18 (28/80), 45 (25/80), 52 (22/80), 58 (21/80), 35 (18/80), 51 (15/80), and 31 (13/80), as shown in Table 2.

**Table 1:** High-risk HPVs and EBV detection in human head and neck cancers.

**Number of samples^#^**	**HPVs+**	**EBV+**	**HPVs+/EBV+**
Positive cases	35/80	41/80	25/80
(%)^##^	(43.7)	(51.2)	(31.2)

*The presence of HPVs and EBV was found in 35 (43.7%) and 41 (51.2%) of the 80 cancer samples, respectively, while we observed that 25 (31.2%) of cancer cases are positive for both high-risk HPVs and EBV. The presence of these oncoviruses was confirmed by PCR and IHC using specific primers for E6/E7 and LMP1 as well as EBNA1 genes of high-risk HPVs and EBV, in addition to monoclonal antibodies for E6 and LMP1, as illustrated in the Materials and Methods section*.

# *The total number of samples examined in this study is 80*.

## *These two methodologies, PCR and IHC, were used to detect the presences of high-risk HPVs and EBV*.

**Table 2:** Presence of HPV types 16, 18, 31, 33, 35, 45, 51, 52, and 58 in human HN cancer.

**No. of cases**	**High-risk HPVs**								
	**16**	**18**	**31**	**33**	**35**	**45**	**51**	**52**	**58**
80	31/80	28/80	13/80	34/80	18/80	25/80	15/80	22/80	20/80

*We note that HPV types 33, 16, 18, and 45 are the most common in HN cancer in the Syrian population*.

**Figure 1:**
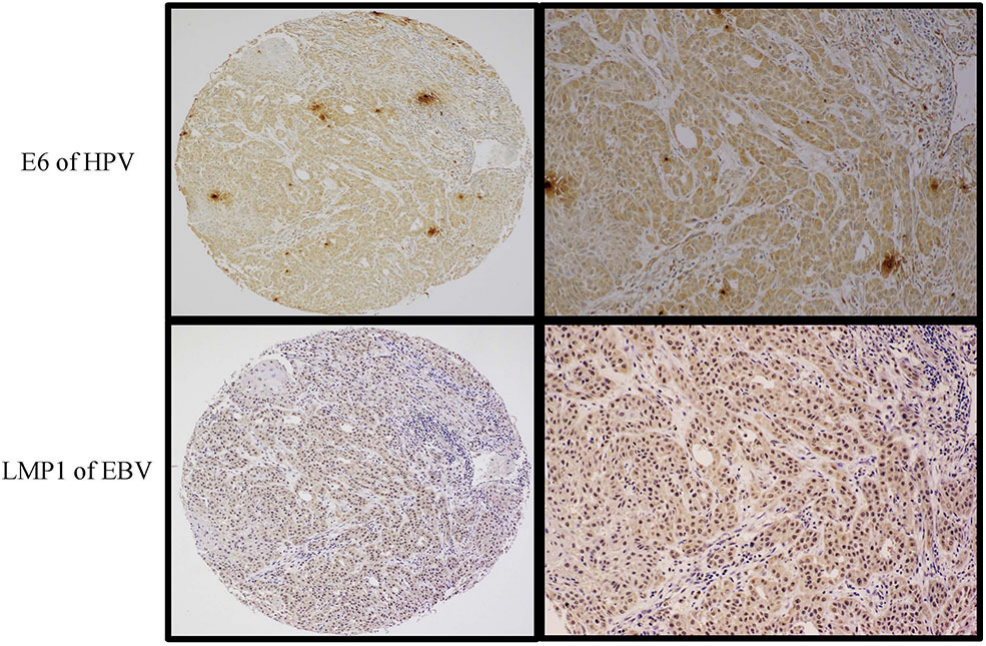
High-grade (Grade 3), non-keratinizing squamous cell carcinomas with basaloid appearance. Tumor cells were diffusely (>75%) and strongly (2+ and 3+ intensity on the scale 0–3) positive for both HPV (E6, upper images) and EBV (LMP1 protein) (lower images). The left-sided images are captured at 4× magnification, while the right-side ones are at 10× magnification (Olympus BX53). Expression of both proteins was assessed by immunohistochemistry using a semiquantitative approach.

**Figure 2:**
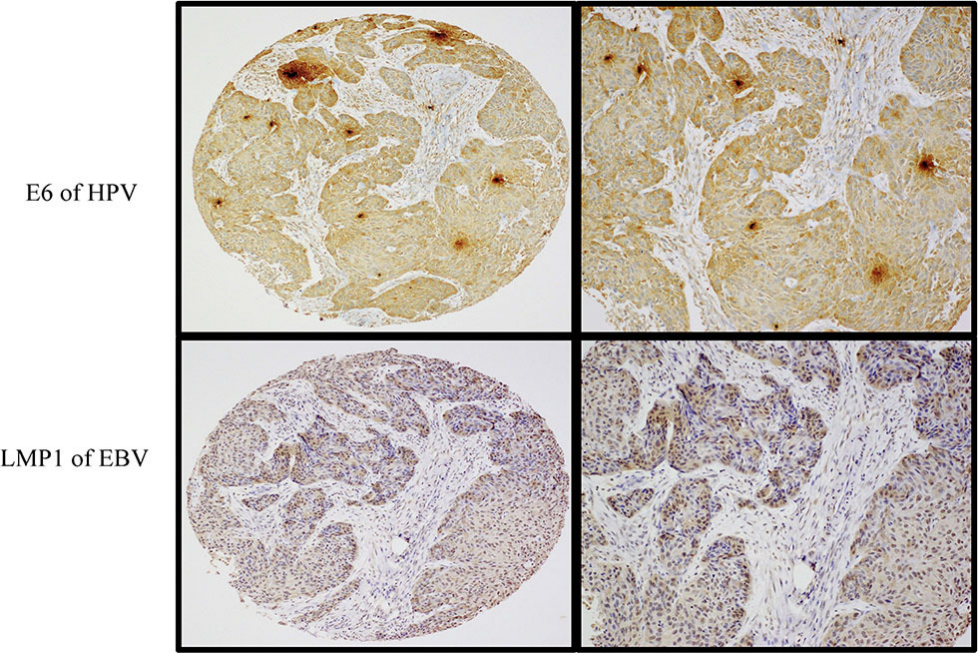
A case of moderately differentiated (Grade 2) squamous cell carcinoma exhibiting co-expression of E6 (upper images) and LMP1 (lower images) oncoproteins of HPV and EBV, respectively. Left-sided images are captured at 4× magnification, while the right-sided ones are at 10× magnification (Olympus BX53). Note that the expression of E6 protein was observed in >90% of cancer cells while LMP1 protein was expressed in ~70% of cancer cells. Expression of both oncoproteins was assessed by immunohistochemistry using a semiquantitative approach.

Next, we investigated the co-presence of high-risk HPVs and EBV in our HN cancer samples by IHC and PCR analysis using monoclonal antibodies, for E6 and LMP1, as well as specific primers for these oncoproteins/genes, respectively; we found that 25 (31.2%) of the 80 cancer cases are positive for both high-risk HPVs and EBV with *P* < 0.001 (Table 1). Furthermore, we examined the relation between the co-presence of these oncoviruses and tumor grade in these samples. Our data revealed that the co-expression of E6 and LMP1 oncoproteins of high-risk HPVs and EBV, respectively, in the majority of cases (88%) is associated with high/intermediate (G3/G2)-grade invasive carcinoma form in comparison with high-risk HPVs+ or EBV+ alone cases or HPVs/EBV-negative cases, which are 4/10 (40%), 5/16 (31.2%), and 3/29 (10.2%) with *P* = 0.00328, 0.00018, and < 0.001, respectively (Table 3 and Figures 1, 2), while it is important to highlight that cancer phenotype in the HPV^+^/EBV^+^ was not linked to a specific HPV type since all our positive cases were infected with more than one type of high-risk HPVs. Finally, normal HN tissues and epithelial cells, which served as controls, were shown to be negative for both high-risk HPVs as well as EBV.

**Table 3:** High-risk HPVs and EBV status in relation to tumor grade in HN cancer samples.

**EBV/HPVs status**	**EBV^**+**^/HPVs^**+**^ (%)**	**EBV^**+**^/HPVs^**−**^ (%)**	**EBV^**−**^/HPVs^**+**^ (%)**	**EBV^**−**^/HPVs^**−**^ (%)**
**TUMOR GRADE**				
High	5 (20.0)	1 (10.0)	2 (12.5)	1 (3.4)
Intermediate	17 (68.0)	3 (30.0)	3 (18.7)	2 (6.8)
Low	3 (12.0)	6 (60.0)	11 (68.7)	26 (89.6)
Number of samples^#^	25 (31.2)	10 (12.5)	16 (20.0)	29 (36.2)

*We notice that 88% of HPVs- and EBV-positive cases are high/intermediate-grade carcinomas in comparison with EBV^+^/HPV^−^, EBV^−^/HPV^+^, and EBV^−^/HPV^−^, which are 40, 31.2, and 10.2% with a P-value of 0.00328, 0.00018, and < 0.001, respectively*.

# *The total number of samples examined in this study is 80*.

*Values inside parentheses denote percentage*.

## Discussion

In this investigation, we explored, for the first time, the incidence/co-incidence of high-risk HPVs and EBV in human HN cancer and the association of their co-presence with tumor grade in Syria, which can also be considered the first study of this type in the ME region. We report that high-risk HPVs and EBV are present in 43.7 and 51.2%, respectively, in our Syrian samples, and the most frequent HPV types in HN cancer in Syria are 33, 16, 18, 45, 52, 58, 35, 51, and 31, correspondingly. Meanwhile, our data pointed out that 31.2% of the samples are positive for both high-risk HPVs and EBV. More significantly, we report that the co-presence of these oncoviruses is associated with high/intermediate tumor grade in 88% of the samples in comparison to HPVs or EBV-positive alone and HPVs/EBV-negative samples. Regarding the most common HPV types in the Syrian population, the present data concur with our previous studies on HPVs in different types of human carcinomas including cervical and breast in Syria where we found that HPV type 33 is the most frequent in these cancer tissues. In this context, HPV type 33 was reported to be the most common in breast cancer in Turkey (30). Herein, it is important to highlight that the Syrian samples were collected from Aleppo province, which is located in the northern part of the country bordering Turkey. Accordingly, our data confirm that specific types of high-risk HPV infection, in human cancers, are related to certain geographic locations, as it was demonstrated by a large number of investigations worldwide (7, 31–36).

Concerning the co-presence of HPVs and EBV in HN cancer in the ME region, Tatli Dogan et al. (37) published a study regarding the incidence of HPVs and EBV in NPCs in Turkey, they found that 72 of their 82 samples are positive for EBV and only one case revealed positive for HPVs; meanwhile, they reported that the highest rate of EBV positivity correspond with undifferentiated NPCs. However, and in agreement with our investigation, one report from North Africa assessed the presence and co-presence of high-risk HPVs and EBV in 70 cases of NPCs from the Moroccan population (38). Their study revealed that 24 of the samples are positive for high-risk HPVs, and the most frequent HPV types are 31, 59, 16, 18, 33, and 35. They found that all their cancer cases are positive for EBV. Consequently, 24 (34%) Moroccan samples were positive for both high-risk HPVs and EBV, which are in their majority NPCs grade III and II. It is important to highlight that the Turkish and Moroccan studies in addition to another investigation from Iran with only 20 cancer cases (39) focused only on the co-presence of HPVs and EBV in NPCs; therefore, our investigation can be considered the first study regarding the co-presence of these oncoviruses in HN cancer in the ME region since our samples include tissues from several HN locations.

On the other hand, in 2012, Jalouli et al. (40) examined the incidence and co-incidence of high-risk HPVs and EBV in 155 oral squamous cell carcinomas (OSCCs) from eight different countries from Europe, Asia, Africa, and North America with a limited number of cases ~20 cancer cases from each country including Sudan and Yemen from the ME region. They found that 35 and 55% of the samples are positive for HPVs and EBV, respectively. They reported that HPVs and EBV are co-present in 21% of all OSCCs. However, no clear conclusions can be drawn from this study due to the limited number of cancer cases from participant countries. Meanwhile, there are two recently published investigations from Europe regarding the presence of HPVs and EBV in human oral cancer; one from Poland showed that HPVs and EBV are co-present in 34.1% of cancer cases in comparison with HPV and EBV infections alone, which are 28.1 and 54.7%, respectively (23). In the second study from Finland, the authors reported that the co-incidence of HPV and EBV is 14% in the population of Finland (41). In comparison, our study focused on the presence/co-presence of HPVs and EBV in HN cancer in Syria with an acceptable number of samples, which allowed us to make an adequate conclusion about these oncoviruses in HN cancer in this country, revealing that 31.2% of the samples are HPVs^+^/EBV^+^, which is comparable with the study published from Poland.

The co-presence of high-risk HPVs and EBV and their association with tumor phenotype in HN cancer is clearly demonstrated in our present study regardless of HPV type since all our HPV/EBV-positive cases are infected with more than one HPV virus strain. Our findings are in agreement with several investigations worldwide, including three from our group in addition to the Turkish and Moroccan studies; data of these reports pointed out that the co-presence of HPVs and EBV is associated with high-grade carcinomas in addition to positive axillary lymph nodes (5, 23, 25–27, 42, 43). Indeed, it has been reported that prevalence of poorly differentiated tumors is four times more frequent in HPV/EBV co-infection in comparison with EBV or HPV infection alone in oral cancer samples from Poland (23); in addition, the study pointed out that there is a significant correlation between tumor dimensions in co-infected patients compared with single infection. However, a recent investigation in NPCs reported that 5-year overall survival is significantly higher in HPV/EBV-positive patients in comparison with HPV/EBV-negative ones (41). Well, this could be due to radiation sensitivity as demonstrated by several investigations. Actually, earlier studies in HPV-positive cases of HNSCC found that the virus takes control of the cellular machinery for DNA repair, altering cell cycle distribution and causing hypoxia during radiation treatment (44, 45). On the other hand, numerous studies on the alteration of radiation response by EBV reported that LMP-1 blocks DNA repair by suppressing the phosphorylation and activity of DNA-dependent protein kinase, a key enzyme of non-homologous end-joining pathway in NPCs, and by repressing ATM, which ultimately modulates resistance of ionizing radiation-induced apoptotic cell death (46).

Apropos the mechanism of HPVs and EBV interaction, based on the fact that high-risk HPVs and EBV oncoproteins share different downstream pathways, we assumed that oncoproteins (E5, E6/E7, LMP1, and EBNA1) of these oncoviruses can cooperate in the initiation and/or progression of several types of human carcinomas where the EMT event can play a crucial role in this procedure (47). Indeed, earlier investigations showed that E5 and E6/E7 oncoproteins of high-risk HPVs can enhance cell invasion and cancer progression via the induction of EMT in several types of human cancers including cervical and oral as well as NPCs (48–53). On the other hand, it has been reported by several investigations that LMP1, LMP2A, EBNA3C, and EBNA1 oncoproteins of EBV can enhance cancer progression via the modulation of EMT in human carcinomas including NPCs (20, 53–56). Meanwhile, our preliminary data showed that E6/E7 of HPV type 16 can cooperate with LMP1 of EBV to enhance EMT progression and consequently cell motility via the activation (phosphorylation) of Erk1/Erk2 and β-catenin (in preparation). Nevertheless, further studies are needed to elucidate the complete pathogenesis and role of the co-incidence of high-risk HPVs and EBV in human carcinomas including HN, especially since HPVs and EBV vaccines are currently available and under clinical trial, respectively (57–59). This is a key step, which could possibly limit HPV and EBV infection and their associated cancers including HN malignancy initiation and development to a metastatic form, thus diminishing cancer-related mortalities especially in developing countries where oncoviruses-associated cancers are still considered major causes of death among both males and females in these countries.

Lastly, with regard to the number of specimens that we were able to amass from Aleppo, Syria, it is essential to confirm our data using a larger number of samples from different areas in this country and in combination with numerous investigations from the ME in general.

## Data Availability Statement

All datasets generated for this study are included in the article/supplementary material.

## Ethics Statement

The studies involving human participants were reviewed and approved by Ethics Committee of the Faculty of Medicine of Aleppo University, # 2009-007. Written informed consent for participation was not required for this study in accordance with the national legislation and the institutional requirements.

## Author Contributions

A-EA, SV, and HA-T conceived the study. LG provided the samples. IG, LG, SV, MM, AY, HA-T, and A-EA analyzed the data. All authors wrote and approved the final version of the manuscript. All authors contributed to the article and approved the submitted version.

## Conflict of Interest

The authors declare that the research was conducted in the absence of any commercial or financial relationships that could be construed as a potential conflict of interest.
